# Implantable CMOS Biomedical Devices

**DOI:** 10.3390/s91109073

**Published:** 2009-11-17

**Authors:** Jun Ohta, Takashi Tokuda, Kiyotaka Sasagawa, Toshihiko Noda

**Affiliations:** 1 Nara Institute of Science and Technology, 8916-5 Takayama, Ikoma, Nara 630-0101, Japan; 2 CREST, Japan Science and Technology Agency, 3-5 Sanban, Chiyoda, Tokyo 102-0075, Japan; E-Mails: tokuda@ms.naist.jp (T.T.); sasagawa@ms.naist.jp (K.S.); t-noda@ms.naist.jp (T.N.)

**Keywords:** biomedical devices, retinal prosthesis, image sensors, CMOS, brain implantation

## Abstract

The results of recent research on our implantable CMOS biomedical devices are reviewed. Topics include retinal prosthesis devices and deep-brain implantation devices for small animals. Fundamental device structures and characteristics as well as *in vivo* experiments are presented.

## 1. Introduction

The trend toward ultra-fine scale in CMOS technologies has not only increased chip integration density but has also enabled the widespread application of CMOS technologies in areas such as image sensors, wireless, micro-electro-mechanical systems (MEMS), and micro total analysis system (μTAS). These features are suitable for biomedical applications. For example, implantable devices require small volume and low power consumption, which are realized by ultra-fine fabrication technology. MEMS are effective technologies for biomedical applications, because MEMS easily form three-dimensional structured which are useful in the applications. Image sensor technology is also very efficient for biotechnology applications in which fluorescence is frequently used for labeling specific cells or detecting neural activity. CMOS technologies are thus highly suitable for implantable devices for biomedical applications and have been used in various types of devices.

In this review, two typical examples of implantable CMOS devices from our research are described: retinal prosthesis devices [1-5] and brain-implantable devices based on CMOS technologies [6-9]. In retinal prosthesis devices, MEMS technologies with CMOS circuits have been used to realize a retinal stimulator based on a microchip array, and in brain-implantable devices, image sensor technology and MEMS have been combined to realize an ultra-compact device to be implanted in the deep brain of experimental small animals. The present review describes the results of recent research on retinal prosthesis devices and brain-implantable devices. In Chapter 2, we discuss the advantages and problems associated with the *in vivo* implantation of CMOS devices. In Chapter 3, we describe retinal prosthesis devices that we have developed. We also describe a multiple microchip architecture that we developed in order to realize a retinal stimulator that uses a large number of stimulus electrodes while bending the stimulator to match the curvature of an eyeball. Chapter 4 describes brain-implantable CMOS imaging devices for measuring the neural activity in the deep brain of a small experimental animal, such as a mouse. Chapter 5 briefly addresses areas for future research and summarizes the present review.

## 2. *In Vivo* Implantation of CMOS Devices

**Figure 1.**
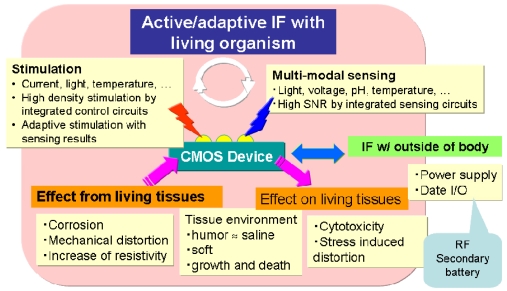
Advantages and problems associated with *in vivo* implantation of CMOS devices.

This section describes the advantages and problems associated with *in vivo* implantation of CMOS devices. Figure 1 summarizes these advantages and problems. The advantages include high performance and versatile functionality in the detection of bio-signals and the stimulation of living cells by on-chip integration of circuits. For example, an on-chip amplifier can detect weak signals with a high signal-to-noise (SN) ratio, and an on-chip multiplexer can be used for multi-site stimulation.

CMOS devices also have the advantage of multi-modal sensing of, for example, physical values such as the amount of light, voltage, and temperature, ions, and chemical entities such as enzymes. CMOS devices also enable multi-modal stimulation such as injection of charge and chemical substances. The important point is that a CMOS device can configure a closed-loop of sense and stimulation. Neural cells can be actively and adaptively stimulated by detecting physical value(s) and analyzing spatio-temporal dynamics. For example, in retinal prosthesis, retinal cell stimulation is often adaptively controlled by monitoring the impedance value. In addition, for deep brain stimulation, such an implanted device is proposed that monitors the amount of dopamine emitted in a patient's brain, determines the optimum value of stimulation, and stimulates the deep brain before the onset of tremor is currently being developed [10]. This is a typical example of a closed-loop device.

Although implanted CMOS devices are expected to provide a highly sophisticated interface with the living body, there are many problems to be solved before realization because both stable and safe operation *in vivo* is required. Figure 1 shows these issues. An implanted device affects living tissues or cells, and these effects can include cytotoxicity and stress-induced distortion. As for cytotoxicity, packaging materials and/or electrode materials may be dissolved into tissue and affect cells. Many discussions including cytotoxicity and mechanical distortions appear in [11]. In addition, in stimulation, electrochemical reactions can occur when the stimulation voltage exceeds the voltage window, and pH changes and/or bubbling can have harmful effects on neural cells [12,13]. Moreover, implanted devices are also affected by the living environment. The living environment is composed primarily of saline solution, so that a CMOS chip with no coating may be damaged. Therefore, a highly water-tight packaging is necessary such as parylene. Of course, this material must be biocompatible. An implanted device is stressed by the living tissues so that it may be distorted or broken. For example, a thinned CMOS chip is easily implanted but may be broken by stress from tissues because Si is fragile when thinned. Living tissues may grow and die, and thus the configuration between the device and tissues may change gradually. This may cause an impedance change between an electrode and living cells. Implanted devices must be designed knowing that the configuration of the device may change.

**Figure 2.**
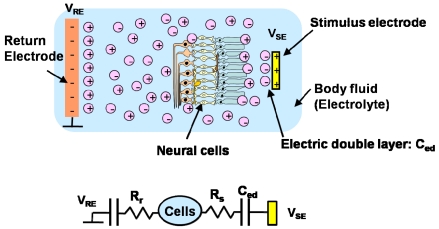
Electrical stimulation of neural cells in body fluid (a) and its equivalent circuits (b).

Next, we consider the stimulation of neural cells in, for example, artificial cochlear and retinal prostheses. In these applications, stimulation is achieved by extra cellular stimulation, in which the potential between the inside and outside of the cell is changed through electrolytes such as a body fluid. Consequently, when a voltage is applied to a stimulus electrode, an electric double layer is produced near the electrode. The thickness of this layer is very thin so that the associated capacitance is large. Resistance also exists in the electrolyte. The equivalent circuits are shown in Figure 2. The impedance between the electrode and the cells may change if the distance between the electrode and the cells changes, as mentioned previously. Stimulation is achieved with a short voltage pulse. This voltage pulse is made biphasic, that is, two consecutive pulses of opposite polarity to achieve charge balance and to deliver a zero net charge into the tissue to ensure long-term safety. In addition, for the purpose of achieving charge balance, a biphasic pulse is usually used to ensure long-term safety. A biphasic pulse consists of two opposite polarity pulses, which deliver no net charges into tissues.

## 3. Retinal Prostheses

### 3.1. Blindness

**Figure 3.**
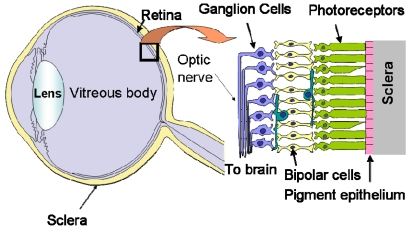
Schematic illustration of the structure of the eye and retina.

As shown in Figure 3, the human retina is a thin, layered tissue with a thickness of 0.1–0.4 mm attached to the inner surface of the eyeball [14]. The retina has a layered structure with photoreceptor cells for light detection in the bottom layer and ganglion cells for output in the top layer. The retina plays an important role in visual information collection and processing, and so dysfunction can result in blindness.

**Figure 4.**
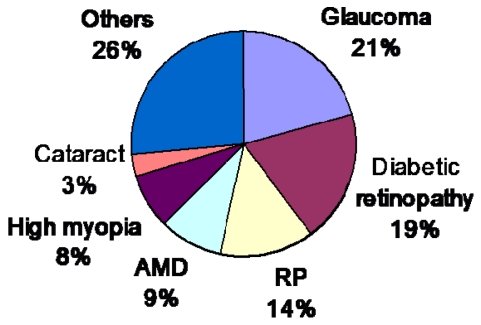
Ratio of causes of blindness in Japan (adapted from [15]). AMD: age-related macular degeneration. RP: retinitis pigmentosa.

Figure 4 shows the ratio of causes of blindness in Japan [15]. Among these diseases, retinitis pigmentosa (RP) and age-related macular degeneration (AMD) have no effective remedies at present. In both cases, the photoreceptors gradually become dysfunctional, and the patient eventually becomes blind. In the United States, the number of patients with AMD has reached 1.7 million, which accounts for the majority of cases of disease-related blindness, and this number increases by some 155,000 every year. Within 25 years, the number of patients with AMD will reach 5.1 million. As such, a cure for AMD must be found as soon as possible.

### 3.2. Principle of Retinal Prosthesis and Types of Stimulation Sites

In RP and AMD, photoreceptor cells are dysfunctional, but most of the other retinal cells, such as ganglion cells, are still alive, unless the disease is in the terminal stage [16,17]. Consequently, by stimulating the remaining retinal cells, visual sensation or phosphene can be evoked. This is the principle of the retinal prosthesis or artificial vision. Based on this principle, a retinal prosthesis device stimulates retinal cells with a patterned electrical signal so that a blind patient may sense a patterned phosphene, or something like an image.

**Figure 5.**
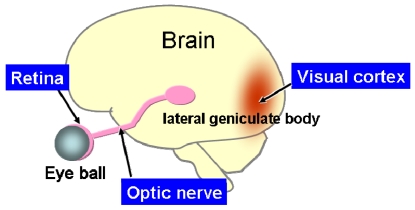
Stimulation sites of the retinal prosthesis.

According to the site at which the retinal stimulator is placed, the retinal prosthesis device is classified into three categories: epi-retinal stimulation [18-21], sub-retinal stimulation [22-25], and suprachoroidal transretinal stimulation (STS) [25,26], which has recently been developed. The stimulation site may be located not only in retinal cells, but also in the pathways to the brain, such as the optic nerves [27], which are the transmission lines of visual information, and, of course, in the visual cortex [28], which is the terminal of the visual information. Figure 5 shows the arrangement of these elements.

In the present review, a prosthesis for which the stimulator is located inside or near the eyeball is referred to as an intraocular retinal prosthesis, and prostheses that involve optic nerve stimulation or visual cortex stimulation, for example, are referred to as extraocular retinal prostheses. These prostheses have certain advantages and disadvantages, which are described below.

#### 3.2.1. Extraocular Retinal Prosthesis

The extraocular retinal prosthesis, which stimulates the visual cortex or optic nerve electrically, can be applied to patients with no retinal cells. This means that these methods can be applied to any disease, including RP and AMD. In the case in which the visual cortex is stimulated, the stimulator is implanted in the surface of the visual cortex by opening a scull. This method has been developed by the Dobelle Institute over a long period of time and has been successfully applied in some patients [28]. The method of stimulating the optic nerve involves covering the optic nerve with a cuff-type electrode to stimulate the nerve [27]. The cuff-type electrodes are illustrated in [29]. Both of these methods require difficult surgical operations because the surgical sites are related to the nervous system or the brain. These methods have only been performed in limited human trials. In addition, these methods must deal with retinotopy, which is the spatial correspondence between the retinal image and the recognition image in the brain. It is difficult to determine the correspondence between the input image and the electrode site on the visual cortex.

#### 3.2.2. Intraocular retinal prosthesis

**Figure 6.**
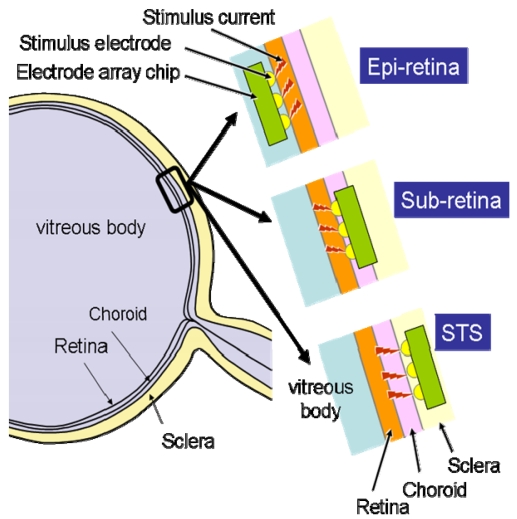
Intraocular retinal prosthesis.

**Figure 7.**
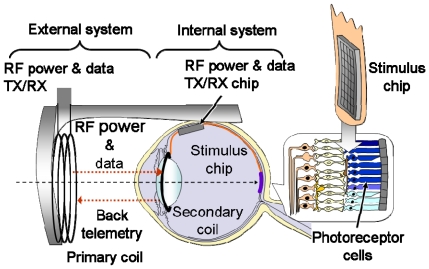
Typical configuration of an intraocular retinal prosthesis system (adapted from [3] © 2009 IEEE).

Stimulation of retinal cells involves an easier surgical procedure and is possibly less affected by retinotopy because the stimulation points are located near the retina. As mentioned previously, this method is classified into three types according to the stimulator implantation site: epi-retinal stimulation [18-21], sub-retinal stimulation [22-24], and STS [25,26]. Figure 6 illustrates these three types of retinal implantation. In these methods, the power supply and stimulus pattern data generated from input image data are transmitted wirelessly by electromagnetic coupling of the primary coil, which is placed outside the body, and the secondary coil, which is placed inside the body, as shown in Figure 7 [3].

These wireless transmission technologies have been established for use in the artificial cochlear system [30]. In Figure 7, the secondary coil is implanted in the lens, but in some cases, it is implanted near the backside of the ear, as in the case of an artificial cochlear system.

## 4. Retinal Stimulator Based On CMOS Technology

### 4.1. Realization of a Large Number of Stimulus Electrodes in a Retinal Stimulator

Several types of intraocular retinal prosthesis developed for use by blind patients have been reported [18-24], although, thus far, with the exception of [22], these prostheses have incorporated only small numbers of electrodes. In order to realize better vision through a retinal prosthesis, over 1,000 electrodes would be preferable. When increasing the number of electrodes, we are faced with problems associated with interconnection between electrodes and external lead wires with good mechanical flexibility. Specifically, the stimulator must be bent to match the curvature of the eyeball.

**Figure 8.**
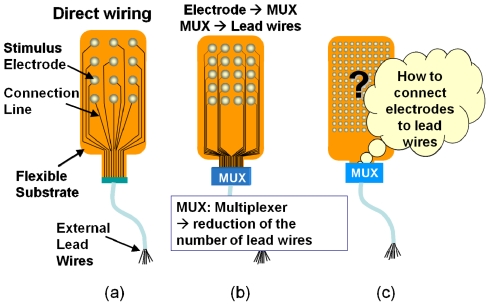
Problem of a large number of electrodes. (a) Direct wiring. (b) Embedding with MUX. (c) Large number of electrodes (adapted from [4]). MUX can reduce the number of external lead wires but not reduce the number of wires from electrodes to MUX.

Figure 8 shows the methods used to realize a stimulus electrode array [4]. A direct connecting method, which is commonly used in retinal prosthesis devices, is shown in Figure 8(a), where each electrode is directly connected by a lead wire. A more sophisticated method is shown in Figure 8(b). In this method, a multiplexer is used to reduce the number of external lead wires. When further increasing the number of electrodes in Figure 8(b), it becomes difficult to connect electrodes to the multiplexer, simply as a result on the increased amount of wiring, as shown in Figure 8(c).

**Figure 9.**
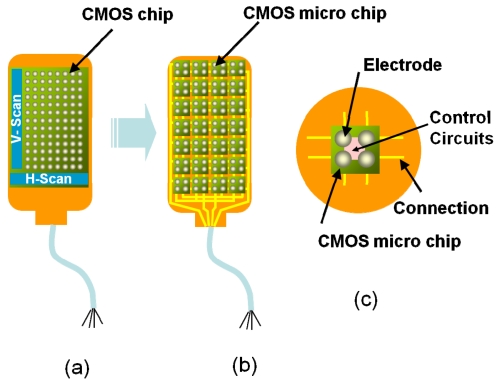
Realization of a large number of electrodes using CMOS technology. (a) CMOS chip. (b) Multiple microchip architecture. (c) Schematic diagram of the microchip (adapted from [4]).

It is a good idea to introduce a CMOS-based chip in the stimulator because scanning circuits (scanner) can be integrated in order to reduce the amount of wiring, as shown in Figure 9(a). Random access can be implemented using decoder circuits instead of scanners. However, it is difficult to use a CMOS chip in a retinal stimulator device, because, for implantation, the CMOS-based stimulator should be thin and flexible in order to fit the eye and to avoid damaging tissue. However, silicon is rigid, and thinning of the CMOS chip increases the risk of breakage.

To solve this problem, we have already developed a new type of smart stimulator that consists of a number of CMOS-based microchips distributed on a flexible substrate, as shown in Figure 9(b) [1,2,4,5]. As shown in Figure 9(c), each microchip incorporates several stimulus electrodes, which can be externally controlled to turn on and off through an external control circuit. In addition to solving the interconnections issue, CMOS-based stimulators offer several advantages, such as signal processing. To allow flexibility, we place several microchips on a substrate in a distributed manner.

The substrate of the stimulator must have both mechanical flexibility and biocompatibility. Among several substrate materials, polymer materials such as polyimide, silicone and parylene are used to meet this requirement [31]. These features protect living tissues from the implanted stimulator, On the other hand, water-resistance is also required for the substrate feature to protect it from the biological environment, Parylene has good water resistant properties, while the others are not completely impervious to water penetration. Since parylene layers are hard to adhere to each other, making it difficult to sandwich metal wire lines in between, parylene is used to cover other substrate materials such as polyimide to protect the substrate from water.

### 4.2. Multiple Microchip Architecture for a Flexible Retinal Stimulator with a Large Number of Electrodes

#### 4.2.1. Device design

**Figure 10.**
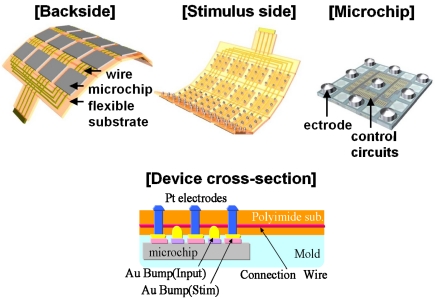
Multiple microchip architecture for a flexible retinal stimulator having a large number of stimulus electrodes.

We have proposed a new structure to realize an array having a large number of electrodes with better extendibility and better sealing characteristics in a biological environment, as compared with the previously proposed stimulator, as shown in Figure 10. This stimulator has been developed primarily for STS, which has been developed recently [25,26], and applied to blind patients. This stimulator can also be applied to other methods, such as sub-retinal stimulation. There are, however, numerous technical challenges that must be overcome when applying CMOS-based stimulators to retinal prostheses, as previously described in Section 2. Next, the device design of the microchip is described.

**Figure 11.**
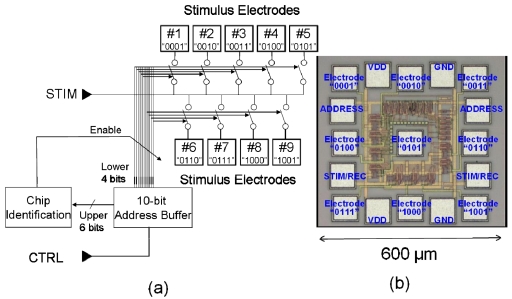
Block diagram (a) and microphotograph (b) of the microchip (adapted from [4]).

The microchip architecture has nine stimulation pads and four input lines, including the power supply lines. A block diagram of the chip is shown in Figure 11(a) [4]. Each stimulation pad is assigned a unique four-bit address that can selectively activate one of the nine electrodes on the microchip. The four input lines are VDD, GND, CTLR, and STIM. The VDD and GND lines are used for the power supply (VDD = 5 V), and control and stimulation can be achieved with only two lines, namely, CTLR and STIM. Each of the stimulation electrodes can be selected with the number of the pulses applied on the CTLR line. This is achieved by the microchip counting the pulses applied on the CTLR line using a 10-bit address buffer. As shown in Figure 11(a), the lower four bits of the address buffer are used for electrode selection, and the upper six bits are used for chip identification. The stimulation current is provided from outside the chip and is fed into the STIM terminal.

One of the stimulation electrodes is selected depending on the value in the lower four bits of the address buffer. The six-bit address space for microchips facilitates the control of an arbitrary number of microchips (up to 64) using only one set of input lines. Consequently, the multi-chip stimulation device platform can configure a 64-chip device with 576 stimulation electrodes. In order to ensure flexibility, the microchip array is assembled at a pitch of 1,000 to 1,200 μm. The microchips are diced from a mother chip, which is fabricated using 0.35 μm standard CMOS technology. The mother chip contains 16 microchips. Figure 11(b) shows microphotographs of a mother chip and a microchip measuring 600 μm × 600 μm.

**Figure 12.**
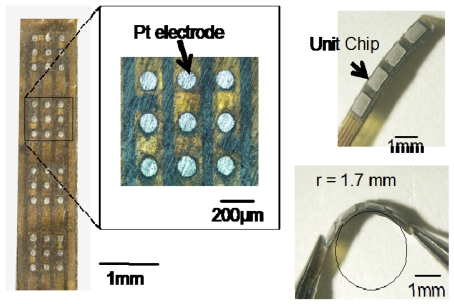
Fabricated retinal stimulator based on the multiple microchip architecture (adapted from [5]).

Figure 12 shows the fabricated retinal stimulator based on the multiple microchip architecture [5]. The four microchips are placed on a flexible polyimide substrate using flip-chip bonding technology. The thickness of the chip is approximately 50 μm. The total thickness of the stimulator is approximately 200 μm. On the front side, nine Pt bulk electrodes are formed on one microchip, so that, in this case, 36 stimulus electrodes are used in the stimulator. The electrode is formed on an Al pad of the microchip using stud bump technology. With the exception of the stimulus electrodes, the surface of the stimulator is covered with epoxy resin or palylene. As shown in Figure 12, the fabricated stimulator can be bent easily, and the radius in this case is approximately the same as that of the rabbit eyeball, *i.e.*, 1.7 mm.

#### 4.2.2. *In vivo* operation

We conducted an *in vivo* experiment in which we implanted the fabricated stimulator into the scleral pocket of a rabbit eye using an operation procedure described in reference [26]. The rabbit was anesthetized. The recording electrode used to measure the electrical evoked potential (EEP) was a stainless-steel screw. The electrode was screwed into the skull at the area of the visual cortex so that the tip touched the dura mater. The reference electrode was screwed into the bregma. The stimulator was inserted into a pocket formed in sclera. A return electrode was inserted into the vitreous cavity. Monophasic 0.5-ms-duration pulses with anodic polarity were used to elicit the EEPs. The pulse used was a single anodic pulse. The responses to 20 to 30 stimuli were averaged for EEP.

**Figure 13.**
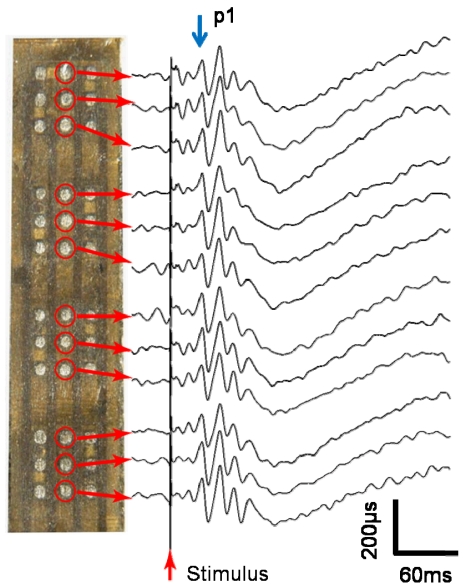
EEP signals obtained in an *in vivo* experiment using the implanted retinal stimulator.

Figure 13 shows the experimental results for the EEPs, where “p1” was concluded to be the EEP signal from the ganglion cells because of the latency of the signal. An anodic pulse was found to be more excited than a cathodic pulse in STS and the discussion of the p1 and other peaks are appeared in [26]. Based on p1, the threshold is approximately 100 μA (<1 mC/cm^2^), which is considered to be adequate for the charge capacity of the electrode (1–4 mC/cm^2^ [31]). For each stimulus electrode, a clear EEP signal was obtained. These experimental results clearly demonstrate that any one electrode among the 36 electrodes can be assigned to be the stimulus electrode, which can be used to stimulate retinal cells.

## 5. *In vivo* CMOS Image Sensing Device for Deep Brain Implantation

### 5.1. Measurement Methods for Neural Activity and Implantable CMOS-Based Imaging Device

**Figure 14.**
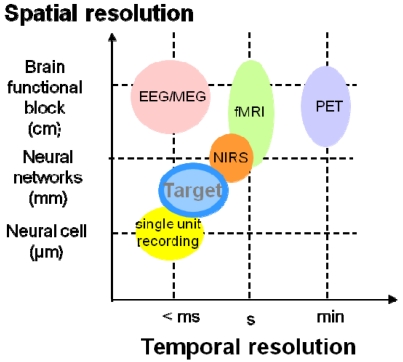
Spatio-temporal map of measurement technologies for neural activity (adapted from [31]).

There is significant demand for devices that can monitor *in vivo* neural activity in the deep brain of small animals, such as the mouse, in real time [32-34]. This requires that the device be able to detect activities with a spatial resolution of less than 1 mm and a temporal resolution of less than 1 sec. However, this is difficult for current measurement methods, such as functional magnetic resonance imaging (fMRI), positron emission tomography (PET), and near infrared spectroscopy (NIRS) to meet both of these requirements, as shown in Figure 14 [31].

We have developed a new CMOS-based imaging device with a sub-mm, sub-second spatio-temporal resolution and have successfully demonstrated monitoring of the time course of serine protease activities inside the mouse hippocampus [6-9]. Through appropriate packaging, the developed device can be used for arbitrary depth imaging inside the mouse brain with minimal impact on brain function. The developed device has functions of imaging and electrical stimulation, which will result in a new bio-imaging tool for neuroscience, medical, and pharmaceutical research.

**Figure 15.**
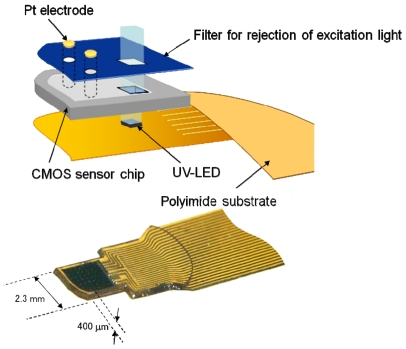
Schematic diagram of an implantable CMOS-based imaging device (adapted from [7]).

We have demonstrated the effectiveness of the device for monitoring the neural activity *in vivo* when the mouse is immobilized [6]. The device is a CMOS-based image sensing chip embedded with electrodes. The chip and excitation UV-LED are placed on a flexible polyimide substrate, as shown in Figure 15. Recently, we have demonstrated the ability to monitor neural activity in the “freely-moving” mouse. In the following section, we briefly summarize the specifications of the developed device. We then show the experimental results of monitoring neural activity in the mouse brain. Finally, a new device that is designed to ensure sufficient fixation in the brain for a freely-moving mouse is presented.

### 5.2. Device Structure and Specifications

The CMOS chip is based on a CMOS image sensor fabricated using standard 0.35 μm CMOS technology. The pixel structure is a three-transistor type active pixel sensor (APS) with a parasitic photodiode composed of n-wells and p-substrate junctions. The specifications of the image sensor are summarized in Table 1.

Figure 15 shows a photomicrograph of the sensor chip [7]. The chip has four stimulus electrode pads (white squares in the figure) and 30 through-holes for excitation light on the surface next to the image sensor pixels.

UV-LEDs can be placed under the through-holes and their emitted light is transmitted through these holes and excited tissues. In some experiments, the UV-LED has been placed next to the chip. The Pt bump can be formed on the electrode pad as a bulk stimulus electrode, as described in Reference [8]. The image sensor surface is coated with a blue filter to suppress the excitation light impinging on the image plane, as mentioned in reference [8]. The total width of the sensor device is approximately 3 mm. The shape of the top of the device is round in order to minimize damage to the tissue during insertion into the brain. The sensor chip and UV-LED are mounted on a polyimide substrate. In the substrate, metal wires are routed from the chip and the UV-LED to an external controller and a power supply. The entire device is covered with an epoxy resin in order to protect the device from the water environment.

### 5.3. In Vivo Experiment Using the Implantable CMOS Imaging Sensor Device

In order to demonstrate the device for functional imaging of the mouse brain, an experiment that involves imaging serine protease activity in the hippocampus was designed and performed. Serine protease is related to memory and learning in the hippocampus. Kainic acid (KA) is a well-known chemical stimulant that can induce the extracellular release of serine protease. In order to detect the activity of serine protease in the hippocampus, synthetic substrate MCA was used. MCA is cleaved by serine protease. The substrate is hydrolyzed due to the presence of the protease, which acts as a catalyst. Once hydrolyzed, the released aromatic amine fluorophore, AMC, fluoresces intensely with a peak wavelength at 470 nm when excited by a UV light source. This fluorescence is used as an indicator of serine protease activity. This process is illustrated in Figure 17.

It is noted that our sensor can detect fluorescence through diffused light. We have already confirmed that the device can detect the diffused light in the depth of about 500 μm [35].

The preparation of the mouse for the surgical procedure and imaging has been reported previously [9]. The packaged device with the attached needle was used in the experiment. Figure 18 shows the position of the device inside the mouse brain.

This substrate solution was used to maximize the available fluorescence signal inside the brain. Fifteen minutes before the substrate pump started, KA was injected intraperitoneally. Figure 19 shows the images captured by the device during the experiment [8]. The fluorescence pattern appears to change dynamically inside the hippocampus. The protease activity at any location in the hippocampus can be determined by plotting the signal level of the pixel at that point. Figure 19(b) shows the activity of two points of interest at coordinates A and B. The onset of protease activity occurs at different times at different locations. At coordinate A, onset occurs approximately 33 min after KA injection, whereas at coordinate B, onset occurs almost immediately after injection of the substrate, 15 min after KA injection.

### 5.4. Measurement of Neural Activity in the Brain of the Freely-Moving Mouse

In order to detect neural activity in the deep brain of the freely-moving mouse, it is necessary to more effectively stabilize the sensor. We are currently developing a new sensor chip, which is shown in Figure 20 [36]. The new sensor is narrower than the previous sensor, which enables the new sensor to be more tightly fixed to the tissue. The fabricated device is also shown in Figure 20. The width of the device is about 2 mm, which is much thinner than that of the previous device. In addition, we are now planning to reduce the number of input/output (I/O) ports in the new sensor to four: the input clock, the output signal, VDD, and GND. The bias voltage can be adjusted after fabrication by trimming the resistor array embedded in the chip. The four bias voltage circuits are used in the chip. The chip specifications are the same as in previous chips, except for the width of the chip and the number of I/Os.

## 6. Conclusions

Implantable CMOS devices are very useful for biomedical applications but there are still a lot of issues to be considered. In this review, we mention two examples of the implantable CMOS devices for biomedical applications; one is s retinal prosthesis and the other is an *in vivo* brain implantable CMOS imaging device.

A retinal stimulator based on multiple microchip based architecture for a large number of stimulus electrodes in STS is described. Better extendibility and reliability are demonstrated, as compared with the previously proposed stimulators. Using the stimulator implanted in a rabbit, the EEP signal has been observed with a threshold of 100 μA.

**Figure 16.**
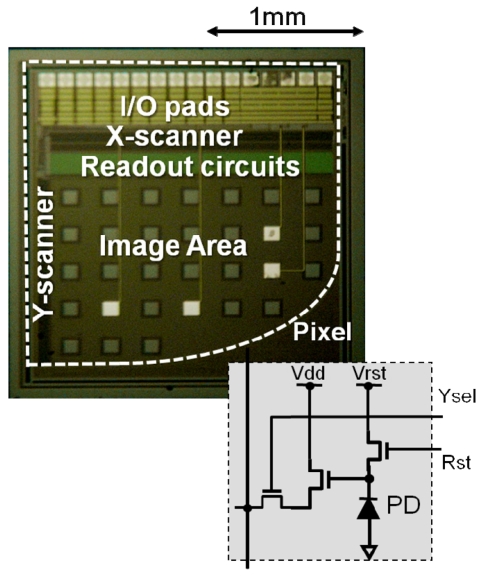
Photomicrograph of the chip. The inset shows the pixel circuit (adapted from [7]).

**Figure 17.**
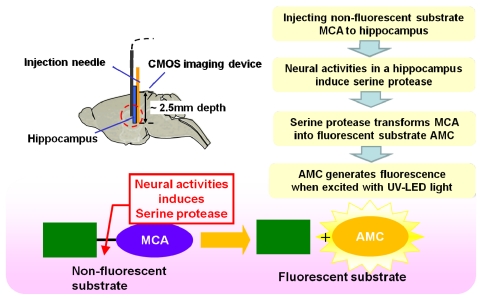
Measurement method of detecting neural activity thorough fluorescence.

**Figure 18.**
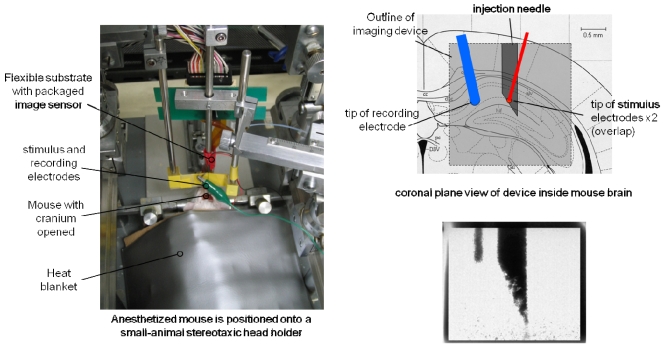
Experimental setup of measuring neural activity in the mouse hippocampus.

**Figure 19.**
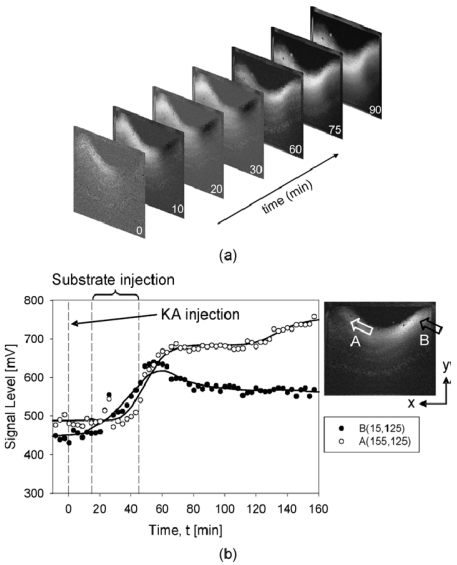
Experimental results of neural activity in the mouse hippocampus obtained using the implanted CMOS imaging device (adapted from [8]). (a) time course of the images obtained, and (b) signal level as a function of time. © 2009 IEEE

**Figure 20.**
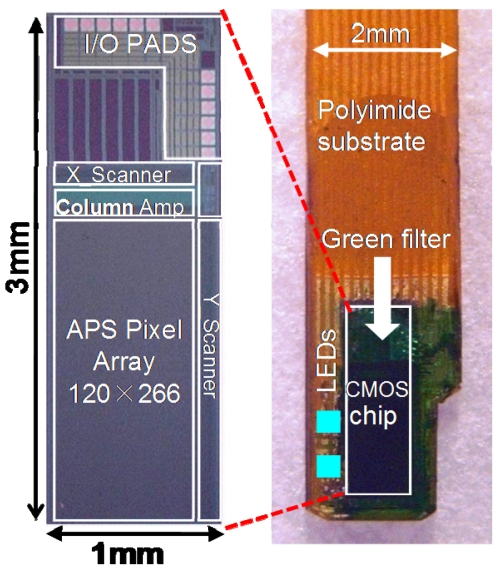
Microphotograph of the sensor chip and the fabricated device implanted into the brain of the freely moving mouse (adapted from [36]). The surface is coated with green filter for GFP (green fluorescence protein). Two LEDs for excitation of GFP are placed on the side of the chip.

The next step in our retinal prosthesis device is to ensure durability and biocompatibility in long term operation inside living tissue. One of the most difficult issues is a package for a Si microchip. Parylene is a good candidate for the package, but for the long term, such as ten years, it is not unknown whether parylene will maintain its the water-resistant characteristics or not. A ceramic hermetic case is effective for water-resistance, but it has a large volume for a microchip. Deposited film of metal is also effective but it is difficult to deposit metal film with no pinholes. If pinholes exist, water can penetrate into the chip. The implantable CMOS image sensing device in the mouse brain is introduced for monitoring the neural activity in the deep brain of a mouse. This demonstrates the effectiveness of the proposed device for use in brain science. The next generation sensor is also shown for freely-moving mouse. The previously-developed device shown in Figure 16 has been demonstrated to operate properly over two weeks. In such short term operation, the UV-LED does not affect tissues, but we need to investigate the effects over longer terms. In addition, we need to exectute the similar test for the newly-developed device shown in Figure 20.

The next step in the implantable CMOS image sensing device is to improve the resolution of the sensor. Bulky optics is not suitable for the implantable device. Microoptics is one of the candidates. The other requirement is to construct a wireless system for a complete implantable device. Although there are a lot of issues to be solved in terms of biocompatibility and durability for a long term operation, implantable CMOS devices will play an important role for the future biomedical applications.

## Figures and Tables

**Table 1.** Specifications of the implantable CMOS imaging chip.

**Technology**	**0.35 μm 2-poly 4-metal standard CMOS**
Power supply voltage	3.3 V
Chip size	2 mm × 2.24 mm
Pixel type	3-Transistor APS
Pixel number	216 × 144
Pixel size	7.5 μm × 7.5 μm
Photodiode	parasitic n-well/p-substrate diode
